# MetaPredictomics: A Comprehensive Approach to Predict Postsurgical Non–Small Cell Lung Cancer Recurrence Using Clinicopathologic, Radiomics, and Organomics Data

**DOI:** 10.1097/RLU.0000000000006086

**Published:** 2025-09-03

**Authors:** Mehdi Amini, Ghasem Hajianfar, Yazdan Salimi, Zahra Mansouri, Habib Zaidi

**Affiliations:** * Division of Nuclear Medicine and Molecular Imaging, Geneva University Hospital, Geneva, Switzerland; † Department of Nuclear Medicine and Molecular Imaging, University of Groningen, University Medical Center Groningen, Groningen, Netherlands; ‡ Department of Nuclear Medicine, University of Southern Denmark, Odense, Denmark; § University Research and Innovation Center, Óbuda University, Budapest, Hungary

**Keywords:** non–small cell lung cancer, PET/CT, radiomics, organomics, deep learning

## Abstract

**Background:**

Non–small cell lung cancer (NSCLC) is a complex disease characterized by diverse clinical, genetic, and histopathologic traits, necessitating personalized treatment approaches. While numerous biomarkers have been introduced for NSCLC prognostication, no single source of information can provide a comprehensive understanding of the disease. However, integrating biomarkers from multiple sources may offer a holistic view of the disease, enabling more accurate predictions. In this study, we present MetaPredictomics, a framework that integrates clinicopathologic data with PET/CT radiomics from the primary tumor and presumed healthy organs (referred to as “organomics”) to predict postsurgical recurrence.

**Patients and Methods:**

A fully automated deep learning-based segmentation model was employed to delineate 19 affected (whole lung and the affected lobe) and presumed healthy organs from CT images of the presurgical PET/CT scans of 145 NSCLC patients sourced from a publicly available data set. Using PyRadiomics, 214 features (107 from CT, 107 from PET) were extracted from the gross tumor volume (GTV) and each segmented organ. In addition, a clinicopathologic feature set was constructed, incorporating clinical characteristics, histopathologic data, gene mutation status, conventional PET imaging biomarkers, and patients’ treatment history. GTV Radiomics, each of the organomics, and the clinicopathologic feature sets were each fed to a time-to-event prediction machine, based on glmboost, to establish first-level models. The risk scores obtained from the first-level models were then used as inputs for meta models developed using a stacked ensemble approach. Questing optimized performance, we assessed meta models established upon all combinations of first-level models with concordance index (C-index) ≥0.6. The performance of all the models was evaluated using the average C-index across a unique 3-fold cross-validation scheme for fair comparison.

**Results:**

The clinicopathologic model outperformed other first-level models with a C-index of 0.67, followed closely by GTV radiomics model with C-index of 0.65. Among the organomics models, whole-lung and aorta models achieved top performance with a C-index of 0.65, while 12 organomics models achieved C-indices of ≥0.6. Meta models significantly outperformed the first-level models with the top 100 achieving C-indices between 0.703 and 0.731. The clinicopathologic, whole lung, esophagus, pancreas, and GTV models were the most frequently present models in the top 100 meta models with frequencies of 98, 71, 69, 62, and 61, respectively.

**Conclusions:**

In this study, we highlighted the value of maximizing the use of medical imaging for NSCLC recurrence prognostication by incorporating data from various organs, rather than focusing solely on the tumor and its immediate surroundings. This multisource integration proved particularly beneficial in the meta models, where combining clinicopathologic data with tumor radiomics and organomics models significantly enhanced recurrence prediction.

Non–small cell lung cancer as a collection of histologic subtypes accounts for ∼85% of lung cancer cases, which holds title for leading cause of cancer mortality.^1^ Surgical resection is the preferred treatment for NSCLC patients, especially in early stages.^2^ Despite great advancements in early diagnosis and treatment of NSCLC, statistics still show decreased quality of life and low survival rate of patients after surgical resection.^3^ Cancer recurrence is the primary cause of treatment failure and death after surgery.^4^ Only 2 years after surgery, depending on the stage of primary cancer, 50%–90% of the patients experience recurrence, and this increases to 90%–95% in 5 years.^5^ Early identification of recurrence risk at the time of diagnosis would enable tailored treatment strategies, potentially leading to improved survival rates.^6^ Tumor-Node-Metastasis staging is the traditional method for treatment response assessment, providing a prognostic framework for clinicians to prognose and adjust treatment options to patients.^3,7^ Within the eighth edition of lung cancer stage classification,^8^ a comprehensive analysis was conducted to enhance the prognostic precision for various stages in patients diagnosed with NSCLC. However, patients with identical disease stages ended up with non-negligible differences in survival, highlighting the insufficiency of relying solely on the TNM staging system as a reliable prognostic marker.^9^ The staggering recurrence rates and consequently low survival outcome of patients demands for novel, more accurate, and comprehensive frameworks for predicting recurrence risk in NSCLC patients.

During the last decade, our interpretation of NSCLC has altered from considering it as an individual lesion, to a disease with a vast range of clinical, genetics, and histopathologic traits, which acquires personalized treatment.^10^ Current research efforts focus on developing prognostic frameworks utilizing machine learning (ML) algorithms due to their unique ability of handling multisourced multidimensional data sets.^11^ By analyzing multiple variables from various sources and learning their complex interrelationships, these methods have the potential to transform current clinical decisions, rooted in established protocols and accumulated experience, into a personalized approach tailored to each individual patient.^12^

Several established sources of information are presently available to unravel different characteristics of NSCLC. Clinicopathologic variables obtained from diverse sources, such as patient’s clinical characteristics, treatment records, histologic and pathologic details of the lesion, pleural, lymphatic, and vascular invasion status, have been the subject of a number of studies to evaluate their prognostic value in NSCLC recurrence.^13–15^ Advancements in omics technologies (genomics, proteomics, etc) have introduced more specific molecular-level variables for predicting NSCLC prognosis.^16^ These variables have been evaluated individually^17,18^ or in combination with clinicopathologic variables toward improved prognostication.^11,19^ The emergence of high-throughput image mining technologies, such as radiomics, opened new opportunities in cancer prognosis by harnessing radiologic data through multidimensional quantitative variables.^20,21^ In NSCLC quantitative prognostication, radiomics expanded imaging applications beyond a limited set of conventional variables, such as tumor standardized uptake value (SUV) metrics from PET scans or shape metrics from anatomic modalities, to high-throughput variables that capture tumor heterogeneities.^22^ The prognostic value of radiomic signatures alone or in combination with TNM stage and other clinicopathologic variables have been evaluated in many studies focusing on NSCLC recurrence and survival prediction.^22–26^

Despite the widespread use of radiomics analysis for NSCLC prognostication, most studies have focused solely on features extracted from the gross tumor volume (GTV).^22,23^ However, it is the spread of the disease into surrounding tissues and the peritumoral environment that largely drives recurrence and poor prognosis.^3^ Several studies incorporated radiomic features extracted from peritumoral environment to enhance the prognostic performance for NSCLC, reporting positive outcomes.^3,27,28^ To predict recurrence-free survival (RFS) of NSCLC patients, Lee et al^27^ developed chest CT-based radiomic models using features from intratumoral and peritumoral regions. Their results showed superiority of features from peritumoral and combined regions over intratumoral for lesions small than 5 cm. Using pretreatment ^18^F-FDG PET images, Mattonen et al^28^ extracted radiomic features from both the metabolic tumor volume (MTV) and a 1 cm extension of penumbra region beyond the tumor surface to predict NSCLC recurrence. The combination of the extracted features with clinical data enhanced prediction performance.^28^ D’Antonoli et al^3^ performed a radiomics analysis on CT-derived peritumoral lung parenchyma within the safe surgical margin of NSCLC tumors, which is considered a 2 cm extension from the tumor contour and presented a nomogram by integrating radiomic and clinicopathologic signatures. Despite the positive results of the abovementioned studies, explored beyond the lesion and its surrounding environment remains outlooked.

Medical images are often acquired from specific body regions, including both the target area and surrounding organs, and in some cases, from the whole body. For NSCLC patients referred to PET/CT imaging, the scan is typically performed from the top of the skull base to the upper thighs to assess potential disease spread.^29^ The metabolic information of different organs reflected in the FDG PET scan, along with corresponding anatomic information from CT, may contain critical information about disease progression and overall organs’ health. Incorporating radiomic features from various organs have been overlooked in previous studies owing to the time-consuming and labor-intensive organs segmentation process. However, advancements in deep learning-based auto-segmentation tools have addressed this limitation.^30–32^ In a previous study by our group, Salimi et al^33^ used DL-driven CT segmentation tool to segment 33 organs from the CT component of PET/CT images, and incorporated the extracted radiomic features from the GTV and presumed healthy organs to predict overall survival in NSCLC patients. The results showed that integrating CT and PET “organomics” significantly improved prognostic performance compared with models only using GTV radiomics.

We believe that none of the many introduced biomarkers can solely provide sufficient information to depict a full picture of NSCLC disease. Only by putting together all these biomarkers, we might gather a holistic view of the disease to enable accurate prognostication. In this study, we present NSCLC Recurrence MetaPredictomics. By utilizing a stacked ensemble learning method, this framework combines patients’ clinicopathologic signature with GTV radiomics and organomics data extracted from preoperative PET/CT images. This comprehensive, multidimensional approach provides a holistic view of patient’s disease, ultimately improving the accuracy of recurrence prognosis.

We believe that no single biomarker, or source of information can provide sufficient data to fully capture the complexity of NSCLC. Only by integrating multiple variables from various sources and unraveling their interrelationships can we achieve a holistic view of the disease that enables accurate prognostication. In this study, we introduce NSCLC Recurrence MetaPredictomics, a framework that utilizes a stacked ensemble learning method to combine patients’ clinicopathologic profiles with GTV radiomics and organomics data extracted from preoperative PET/CT images. This comprehensive, multidimensional approach offers a more complete understanding of the patient’s disease, ultimately enhancing the accuracy of recurrence prognosis.

## PATIENTS AND METHODS

**FIGURE 1:**
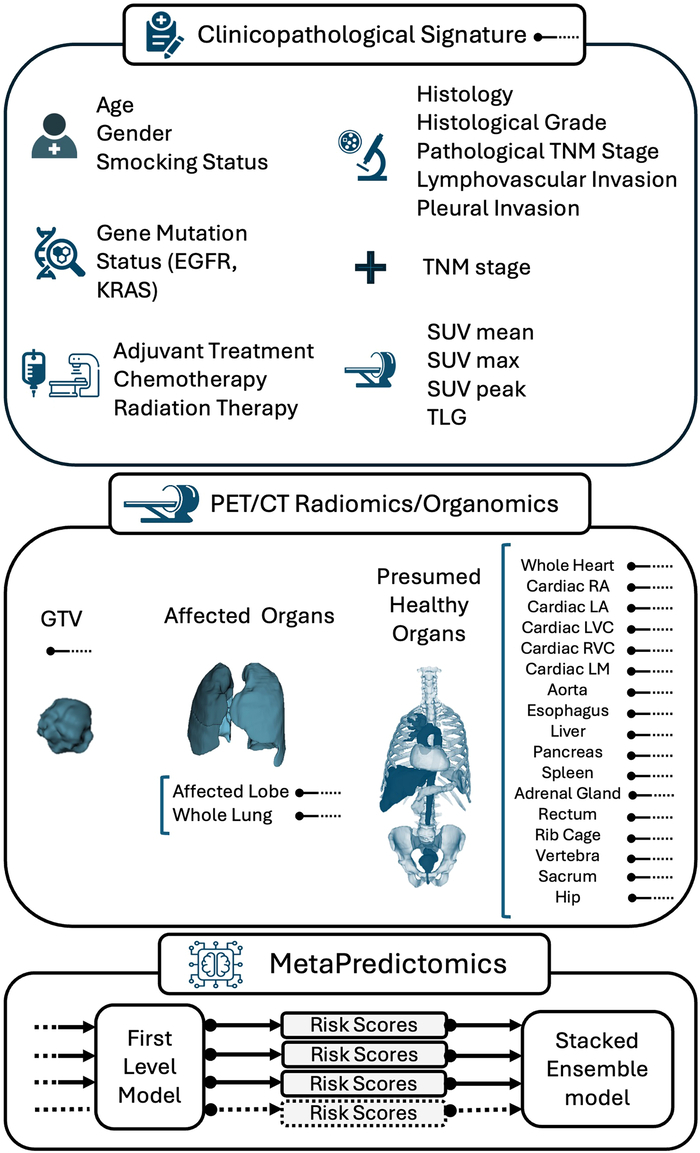
The proposed MetaPredictomics framework for predicting NSCLC recurrence.

Figure 1 illustrates the proposed MetaPredictomics framework for predicting NSCLC recurrence. This framework integrates various clinical data (patient’s characteristics, histopathologic data, genes mutation status, etc) gathered through NSCLC standard-of-care procedures into a conventional clinicopathologic signature. This clinicopathologic signature combines high-throughput PET/CT imaging biomarkers (radiomics) obtained from the GTV and affected and healthy organs. This comprehensive, multidimensional approach offers a holistic view of patient’s disease, ultimately improving the recurrence prognostic accuracy. The following sections provide a detailed explanation of the data set used and the proposed framework.

### Data Set

**FIGURE 2:**
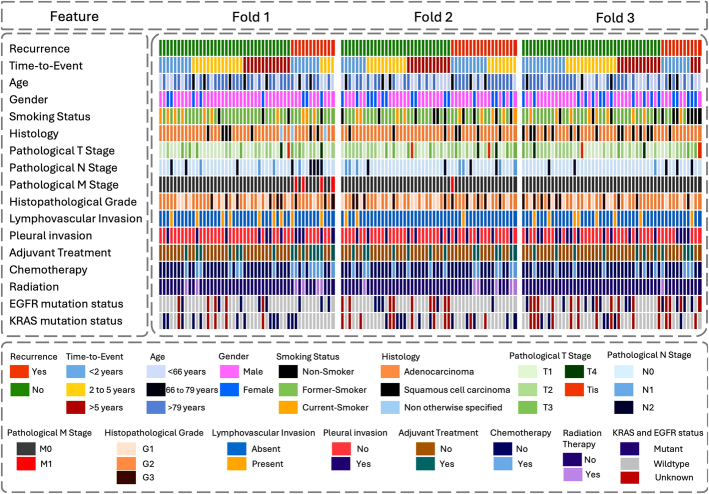
The clinical information of patients as 3 heatmaps for the 3 data set folds.

In this study, we adopted “NSCLC RadioGenomics dataset” from the open-access repository *“The Cancer Imaging Archive (TCIA).”* ^34^ A total of 211 NSCLC patients referred for surgical treatment (2008–2012) were included. Patients underwent ^18^F-FDG PET/CT imaging before surgery, and their clinical characteristics and treatment history were recorded. Sample tissues were obtained from excised tumors and analyzed to provide histopathologic as well as gene mutation information. Patients were then followed up postsurgery for recurrence and survival. Not all the information was available for every patient. We avoided any imputation and enrolled 145 patients having both imaging and clinicopathologic data fully available. The Kaplan-Meier curves of overall survival of the patients with and without recurrence were plotted, and log-rank test was performed between the groups. We divided the data set into 3 folds while preserving the number of recurrence events within each fold. These 3 folds were used for the rest of the study to train and evaluate all models in a 3-fold cross-validation manner. The recurrence-free-survival (RFS) Kaplan-Meier curves of the 3 folds were plotted, and log-rank tests were applied to check for significant RFS differences between the folds. Toward clear presentation of the data set in use, the clinical information of all patients is illustrated as heatmaps in Figure 2. PET/CT acquisition protocols and reconstruction characteristics are listed in Table 1.

**TABLE 1:** PET/CT Acquisition Protocols and Reconstruction Characteristics of the Used Data Set

	Fold 1	Fold 2	Fold 3
CT			
Manufacturer	GE [44], SIEMENS [1], Philips [3]	GE [41], SIEMENS [4], Philips [3]	GE [44], SIEMENS [5]
kVp (V)	126±9.1	126±9	127±9.6
Tube current (mA)	111.5±91.9	114±74.73	93.102±47.283
Pixel spacing (mm)	0.979±0.614	0.998±0.0874	0.985±0.055
Slice thickness (mm)	3.984±0.488	4.011±0.515	3.872±0.398
Pitch factor	1.067±0.218	1.053±0.231	1.0529±0.214
PET			
Injected dose (Mbq)	480±100	471±90.22	480±99.91
Pixel spacing (mm)	4.436±0.795	4.419±0.749	4.375±0.782
Slice thickness (mm)	3.454±0.402	3.526±0.505	3.486±0.517
Reconstruction algorithm	OSEM [7], 3D IR [23], VPHD [4], RAMLA [3], VPFX [4], VPHDS [7]	OSEM [8], 3D IR [22], VPHD [2], RAMLA [3], VPFX [4], VPHDS [6], PSF [2]	OSEM [6], 3D IR [24], VPHD [6], VPFX [3], VPHDS [7], PSF [3]
Scatter correction method	Model-based [39], SS-SIMUL [3], Convolution Subtraction [6]	Model-based [37], SS-SIMUL [3], Convolution Subtraction [7]	Model-based [45], Convolution Subtraction [4]

### Gross Tumor Volume and Organ Segmentation

#### Organ Segmentation

A CT-based organ auto-segmentation tool previously developed in our center^35^ was used to delineate 19 organs from the CT scans of the used data set. The segmentation tool is a deep learning-based framework using a nnU-Net network.^36^ The segmentation model was trained/validated in a 5-fold cross-validation setting, with the final model obtained by ensembling all folds inferenced on the CT component of our data set to extract the organs’ masks. Table 2 lists the segmented organs classified as affected and healthy. Organs are also classified according to tissue type into soft, lung, and bone tissues. To identify the affected lung lobe, 5 lobes, namely left lower lobe (LLL), right lower lobe (RLL), right middle lobe (RML), left upper lobe (LUL), and right upper lobe (RUL), were delineated, and the lobe containing the biggest portion of the GTV was selected. The segmentations of organs were visually checked for any significant errors.

**TABLE 2:** The List of Segmented Organs, Categorized to Affected or Presumed Healthy Organs, Also Classified Based on the Tissue Composition of the Organ

	Tissue Type	Organ
Affected organ	Lung tissue	Affected lung lobe
		Whole lung
Healthy organ	Soft tissue	Whole heart
		Cardiac right artrium
		Cardiac left artium
		Cardiac left ventricle cavity
		Cardiac right ventricle cavity
		Cardiac left myocardium
		Aorta
		Esophagus
		Liver
		Pancreas
		Spleen
		Adrenal glands
		Rectum
	Bone tissue	Rib cage
		Vertebra
		Sacrum
		Hip

#### Gross Tumor Volume Segmentation

GTV masks of the CT image were available as the segmentation was performed manually by experts in previous studies conducted by our group on the same data set.^23,25^ Toward fully automated frameworks, a deep-learning-based model was developed previously in our group^33^ to segment NSCLC tumors from CT images. Using 3 public data sets and a nnU-Net pipeline, Salimi et al^33^ trained a deep learning-based segmentator that achieved an average Dice coefficient of 0.92 ± 0.08 on the same data set used in this study. However, as the GTV masks manually delineated by physicians were available to us, we did not use the automated model in this study.

### Feature Sets Establishment

#### Clinicopathologic Signature

This signature includes various biomarkers collected at different stages of the clinical route for NSCLC management. It encompasses patient characteristics, such as age, sex, and smoking status, information obtained from analysis of surgically excised samples, including histology, histologic grade, lymphovascular and pleural invasion status, KRAS and EGFR mutation status, pathologic T, N, and M stages, conventional image-derived PET biomarkers, such as SUV_mean_, SUV_max_, SUV_peak_, and total lesion glycolysis (TLG), and the patient’s treatment history, including chemotherapy, adjuvant therapy, and radiation therapy.

#### Radiomic Feature Sets

PyRadiomics (version 3.0.1) was used to extract radiomic features from PET and CT images of the NSCLC GTV, as well as from the patient’s affected and healthy organs. A total of 107 features were extracted from each region of interest (ROI), including 19 statistical first-order, 26 morphologic (16 3D-based and 10 2D-based), 24 gray-level co-occurrence matrix (GLCM), 16 gray-level run-length matrix (GLRLM), 16 gray-level size zone matrix (GLSZM), 5 neighboring gray-tone difference matrix (NGTDM), and 14 gray-level dependence matrix (GLDM) features. CT and PET features were pooled to form a feature set (including 2×107=214) for each of the organs and the GTV. CT images were resampled to 1×1×1 mm^3^ isotropic voxel space, and gray value discretization with a fixed bin width of 10 Hounsfield Units (HUs) was applied. The intensity ranges for CT images were clipped as follows: (−1000, 1000) HUs for GTV feature extraction, (−1000, 100) HUs for organs containing lung tissue, (−500, 500) HUs for soft-tissue organs, and (0, 1000) HUs for organs with bony structures. PET images were resampled to 3×3×3 mm^3^ isotropic voxel space. Images were converted to SUV, and a gray level discretization with a fixed bin width of 0.1 SUV was applied, after clipping the intensities to (0, 30) SUVs.

### Recurrence Prognostic Model

To integrate information from multiple sources, we implemented a stacked ensemble learning framework. In this approach, a first-level time-to-event model is applied to each of the defined feature sets. Each model outputs risk scores associated with the patients. These risk scores are then used as input features for a second-level model, or “meta-model,” to predict the time to recurrence.

#### First Level Models

A 3-fold cross-validation setting was utilized for each input feature set. Feature values of the patients in the 2 training folds were pooled and normalized using z-score method, then mean and SD values (from training set) were used to normalize the features in the test fold. The first-level models consisted of a feature selection process followed by a time-to-event analysis machine. For feature selection, a Spearman correlation test was first applied to feature pairs, removing redundant features (one feature from each pair with 𝑅^2^ > 0.9 was excluded). Next, a univariate Cox proportional hazards (CoxPH) model was fitted to each feature using 100 bootstraps (on the training folds) to assess features relevance. Features were ranked by their average concordance index (C-index), and the top 10 with the highest performance were selected. For time-to-event analysis, we used a Glmboost model,^37^ where a generalized linear model is fitted using a boosting algorithm that operates on component-wise univariate linear models. The trained Glmboost model was applied on the test fold with 1000 bootstraps, and the performance was assessed by the average C-index (1000 for each test fold).

#### Stacked Ensemble Hazard Model

In the second stage, we implemented stacked ensemble hazard modeling by using the risk scores from the first-level models. Only models with an average C-index ≥0.6 across the 3 folds were used in the stacked ensemble models. A grid search was performed to explore different combinations of the input models to identify the optimal set for the meta-model. The meta-model also used a Glmboost algorithm. This process was carried out using the same 3-fold cross-validation used for first-level models.

#### Kaplan-Meier Analysis

The first-level models and the best meta-model were evaluated using Kaplan-Meier curves. The median risk score obtained from patients in the test folds was used to categorize patients into 2 groups: low risk and high risk. A log-rank test was conducted to compare the 2 groups, using a significance level of *P* <0.05.

#### Nomogram and Calibration

A prognostic nomogram was constructed based on the best model. The nomogram was developed to estimate individualized recurrence probabilities at 1, 3, and 5 years. Calibration of the model was evaluated by plotting predicted recurrence probabilities against observed outcomes estimated through Kaplan-Meier analysis. Patients were stratified into 5 quantile-based risk groups, and 1000 bootstrap resamplings were performed to adjust for optimism. Calibration plots for 1, 3, and 5-year survival were generated to assess the agreement between predicted and actual recurrence probabilities.

#### Performance Comparison

The performances of the models were compared using the Mann-Whitney *U* test applied to their C-index distributions across bootstrap iterations. The resulting *P-*values were corrected for multiple comparisons using the Benjamini-Hochberg procedure to control the false discovery rate (FDR), and *P*-values below 0.05 were considered as significant.

## RESULTS

### Demography and Organ Segmentation

The clinical information of all patients is illustrated in Figure 2 as 3 heatmaps according to the data set folds. Table 2 lists the PET/CT acquisition and reconstruction characteristics. From the 145 enrolled patients, 41 experienced recurrences during their follow-up time. Supplemental Figure 1A (Supplemental Digital Content 1, http://links.lww.com/CNM/A575) shows the Kaplan-Meier curves for overall survival of patients with recurrence versus without recurrence, showing significant log-rank test *P*-value <0.001. Supplemental Figure 1B (Supplemental Digital Content 1, http://links.lww.com/CNM/A575) shows the RFS Kaplan-Meier curves for patients in the 3 folds. The log-rank tests between these 3 curves showed nonsignificant RFS differences between data set folds with *P*-values all above 0.05.

**FIGURE 3:**
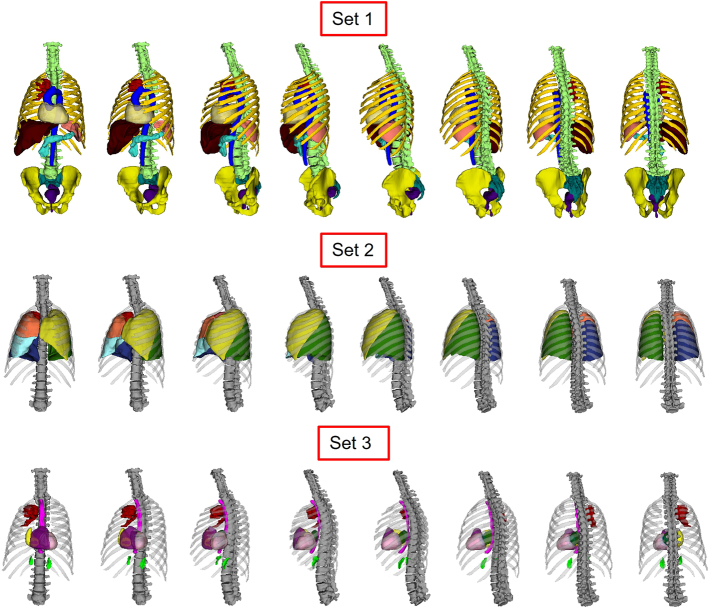
Example of organs segmented on the CT image of a patient in 3D visualization. Set 1 shows the vertebra, rib cage, sacrum, hip, whole heart, aorta, liver, pancreas, spleen, rectum, and GTV. Set 2 includes the rib cage and vertebra, lung lobes, and GTV. Set 3 shows rib cage and vertebra, esophagus, heart substructures, adrenal glands, and the GTV.

An example of organs segmented from CT images of a patient (from the PET/CT scan) is shown in Figure 3. The organs are shown in 3 sets to avoid overlap. The performance of the used segmentation model for each organ is presented.^35^

### Models Performance

Table 3 presents the results of the first-level models with 95% CI for each test fold as well as the average value of the 3 test folds. The clinical model showed the best performance with an average C-index of 0.67 over the 3 external folds. Following the clinical model, GTV and the whole-lung models achieved the highest performance with C-index of 0.65. The affected-lung-lobe model achieved slightly lower performance with C-index of 0.63. Among the healthy organs, aorta model achieved the best performance with average C-index of 0.65, followed by esophagus and adrenal glands models achieving an average C-index of 0.63. Fourteen models achieved an average C-index (over 3 test folds) equal or higher than 0.6, which were used as inputs for the meta (stacked ensemble) model.

**TABLE 3:** The Results of the First-level Models for Each Test Fold as Well as the Average Value of the 3 Test Folds in Concordance Index (C-index)

	Model	Fold 1	Fold 2	Fold 3	Average
	Clinicopathologic	0.74 (0.73–0.74)	0.69 (0.68–0.69)	0.57 (0.57–0.57)	0.67 (0.66–0.67)
	GTV	0.71 (0.71–0.72)	0.62 (0.62–0.63)	0.61 (0.61–0.62)	0.65 (0.64–0.65)
Affected organs	Affected lung lobe	0.66 (0.66–0.67)	0.63 (0.62–0.63)	0.59 (0.59–0.6)	0.63 (0.63–0.63)
	Whole lung	0.72 (0.72–0.73)	0.63 (0.63–0.64)	0.61 (0.61–0.62)	0.65 (0.65–0.66)
Healthy organ	Whole heart	0.61 (0.6–0.61)	0.6 (0.59–0.6)	0.6 (0.6–0.61)	0.6 (0.6–0.61)
	Cardiac right artrium	0.58 (0.58–0.58)	0.56 (0.56–0.56)	0.59 (0.59–0.59)	0.58 (0.58–0.58)
	Cardiac left artium	0.58 (0.58–0.59)	0.55 (0.55-0.56)	0.6 (0.6–0.61)	0.58 (0.58–0.58)
	Cardiac left ventricle cavity	0.65 (0.65–0.66)	0.57 (0.57–0.58)	0.59 (0.59–0.6)	0.61 (0.6–0.61)
	Cardiac right ventricle cavity	0.6 (0.59–0.6)	0.6 (0.6–0.61)	0.6 (0.6–0.6)	0.6 (0.6–0.6)
	Cardiac left myocardium	0.59 (0.59–0.59)	0.57 (0.56–0.57)	0.61 (0.6–0.61)	0.59 (0.58–0.59)
	Aorta	0.67 (0.67–0.68)	0.58 (0.58–0.59)	0.68 (0.67–0.69)	0.65 (0.64–0.65)
	Esophagus	0.62 (0.61–0.62)	0.65 (0.64–0.65)	0.63 (0.63–0.63)	0.63 (0.63–0.63)
	Liver	0.67 (0.67–0.68)	0.58 (0.58–0.59)	0.59 (0.59–0.6)	0.62 (0.61–0.62)
	Pancreas	0.58 (0.58–0.59)	0.62 (0.62–0.63)	0.62 (0.61–0.62)	0.61 (0.6–0.61)
	Spleen	0.59 (0.58–0.59)	0.61 (0.61–0.62)	0.59 (0.59–0.59)	0.6 (0.59–0.6)
	Adrenal glands	0.62 (0.61–0.62)	0.57 (0.57–0.57)	0.69 (0.69–0.7)	0.63 (0.62–0.63)
	Rectum	0.57 (0.57–0.58)	0.56 (0.56–0.57)	0.6 (0.6–0.61)	0.58 (0.58–0.58)
	Rib cage	0.61 (0.6–0.61)	0.56 (0.56–0.56)	0.6 (0.6–0.61)	0.59 (0.59–0.59)
	Vertebra	0.58 (0.58–0.59)	0.56 (0.56–0.56)	0.62 (0.62–0.63)	0.59 (0.62–0.63)
	Sacrum	0.62 (0.62–0.63)	0.6 (0.59–0.6)	0.62 (0.62–0.63)	0.62 (0.61–0.62)
	Hip	0.56 (0.56–0.57)	0.57 (0.57–0.58)	0.59 (0.58–0.59)	0.58 (0.57–0.58)

The range of average C-index (over 3 test folds) in the top 100 meta models was 0.731 (first model) to 0.704 (100th model). Table 4 reports the performance of the top 10 meta-models with their 95% CI sorted by their average C-index over the 3 test folds. The best meta-model was from the combination of clinical + GTV + whole lung + esophagus + pancreas, achieving an average C-index of 0.731 over the 3 test folds. The best performance over one single external fold was obtained from the second meta-model (clinical + GTV + whole lung + sacrum) with fold 1 as the external fold, which achieved a C-index of 0.816.

**TABLE 4:** The Performance of the Top 10 Meta-models Sorted by Their Average C-index Over the 3 Test Folds

	Models	Fold 1	Fold 2	Fold 3	Average^*^
#1	Clinical+GTV+whole lung+esophagus+pancreas	0.80 (0.79–0.8)	0.69 (0.69–0.69)	0.71 (0.7–0.71)	0.731 (0.73–0.74)
#2	Clinical+GTV+whole lung+sacrum	0.82 (0.81–0.82)	0.64 (0.64–0.65)	0.74 (74–0.74)	0.731 (0.73–0.74)
#3	Clinical+GTV+whole lung+pancreas+sacrum	0.82 (0.82–0.82)	0.64 (0.64–0.64)	0.73 (0.73–0.74)	0.730 (0.73–0.73)
#4	Clinical+affected lung lobe+whole lung+esophagus+pancreas	0.76 (0.76–	0.73 (0.72–0.73)	0.70 (0.7–0.71)	0.729 (0.73–0.73)
#5	Clinical+whole lung+esophagus	0.78 (0.78–0.79)	0.73 (0.73–0.74)	0.67 (0.67–0.68)	0.729 (0.73–0.73)
#6	Clinical+GTV+affected lung lobe+whole lung+esophagus+pancreas	0.79 (0.79–0.8)	0.69 (0.68–0.69)	0.71 (0.7–0.71)	0.729 (0.73–0.73)
#7	Clinical+whole lung+esophagus+pancreas	0.76 (0.75–0.76)	0.72 (0.71–0.72)	0.71 (0.71–0.72)	0.727 (0.73–0.73)
#8	Clinical+GTV+affected lung lobe+whole lung+esophagus+liver+pancreas	0.79 (0.79–0.8)	0.69 (0.69–0.7)	0.70 (0.7–0.71)	0.727 (0.73–0.73)
#9	Clinical+GTV+whole lung+esophagus+liver+pancreas	0.80 (0.79–0.8)	0.69 (0.69–0.69)	0.70 (0.7–0.71)	0.726 (0.72-–0.73)
#10	Clinical+GTV+affected lung lobe+whole lung+sacrum	0.79 (0.79–0.79)	0.63 (0.63–0.64)	0.76 (0.75–0.76)	0.76 (0.72–0.73)

Result of each fold is as well presented.

^*^ All values are reported with 2 decimal places, except for the average results, which are shown with 3 decimal places to highlight subtle differences.

**FIGURE 4:**
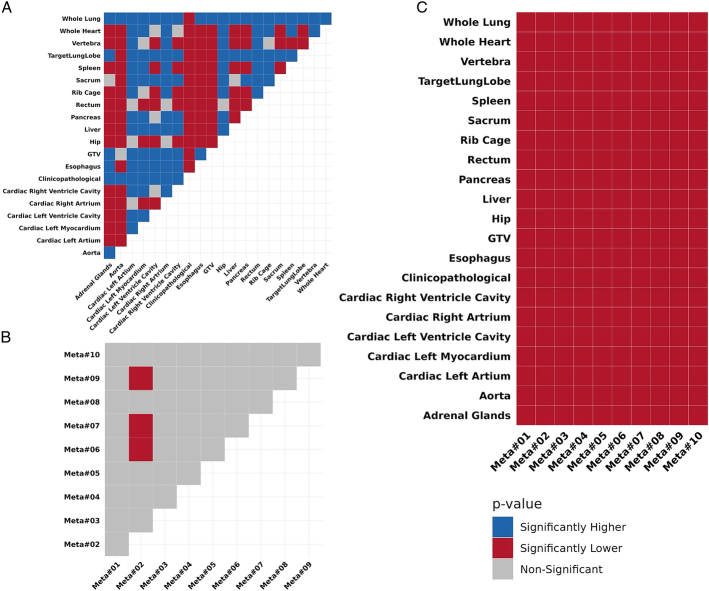
Statistical comparison of model performance based on their resulting C-index values across bootstrap iterations. The results are shown for comparison of models on the rows against models on the columns. **A,** Pairwise comparisons among first-level models. **B,** Comparison of the top 10 meta-models. **C,** Comparison of meta-models with first-level models.

Figure 4 presents the statistical comparison of model performances. Panel A displays pairwise comparisons among the first-level models. The clinicopathologic model demonstrated significantly superior performance compared with all others. The whole-lung model also showed strong performance, outperforming all models except the clinicopathologic model. Panel B compares the top 10 meta-models. Most meta-models exhibited comparable performance, except for Meta#02, which significantly outperformed Meta#06, Meta#07, and Meta#09. Panel C compares each meta-model against the first-level models. All meta-models significantly outperformed every first-level model.

**FIGURE 5:**
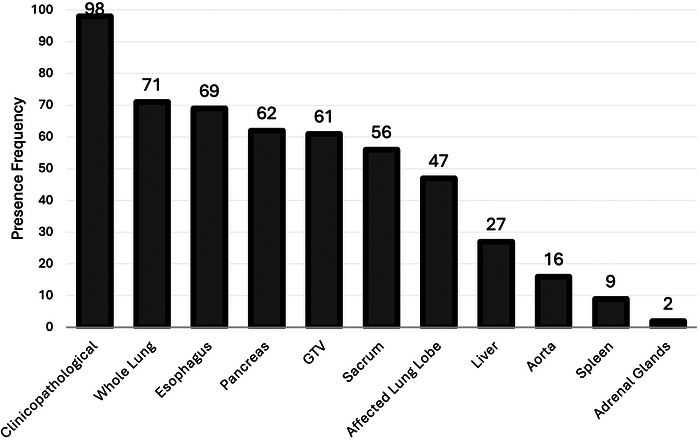
The frequency of the presence of first-level models in the top 100 meta (stacked ensemble) models.

Figure 5 shows the frequency of the first-order models in the top 100 meta-models. Five most repeated models were clinical model with 98/100 repetitions, whole lung with 71/100, esophagus with 69/100, pancreas with 62/100, and GTV with 61/100. First-order models present in the top 100 meta-models (models appeared in Figure 5) were considered for further analysis.

**FIGURE 6:**
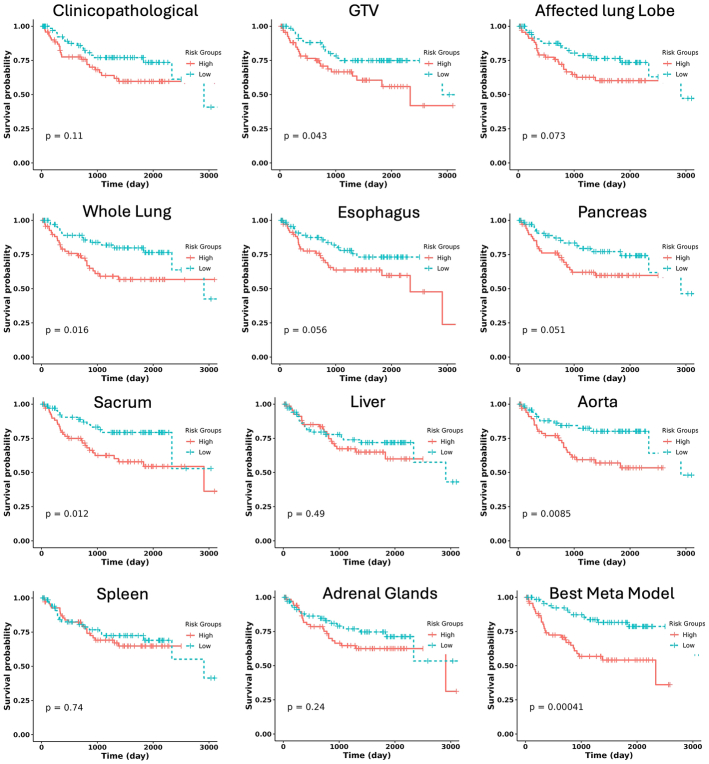
Kaplan-Meier curve of the first-order model as well as the best meta (stacked ensemble) model. The median risk score obtained from patients in the test folds was used to categorize patients into low and high-risk groups. A log-rank test was conducted to compare the 2 groups using a significance level of *P* <0.05.

Figure 6 shows the Kaplan-Meier curve of the first-order models (only models present in the top 100 meta-models) as well as the best meta-model, with the log-rank *P*-values for dichotomizing patients into low and high-risk groups. GTV, whole lung, sacrum, and aorta had significant log-rank *P*-values. The best meta-model had the lowest significant *P*-value (0.00041).

A nomogram was developed based on the meta#1 model to enable individualized prediction of 1, 3, and 5-year recurrence probabilities. Calibration plots were also generated to assess the agreement between predicted and observed recurrence outcomes. The nomogram and corresponding calibration plots are provided in Supplemental Figures 2 and 3 (Supplemental Digital Content 1, http://links.lww.com/CNM/A575). respectively. As shown in the calibration plots, the predicted probabilities demonstrate good agreement with the actual outcomes for the 1-year and 2-year recurrence prediction, while this degraded for the 5-year recurrence prediction.

### Selected Features

**FIGURE 7:**
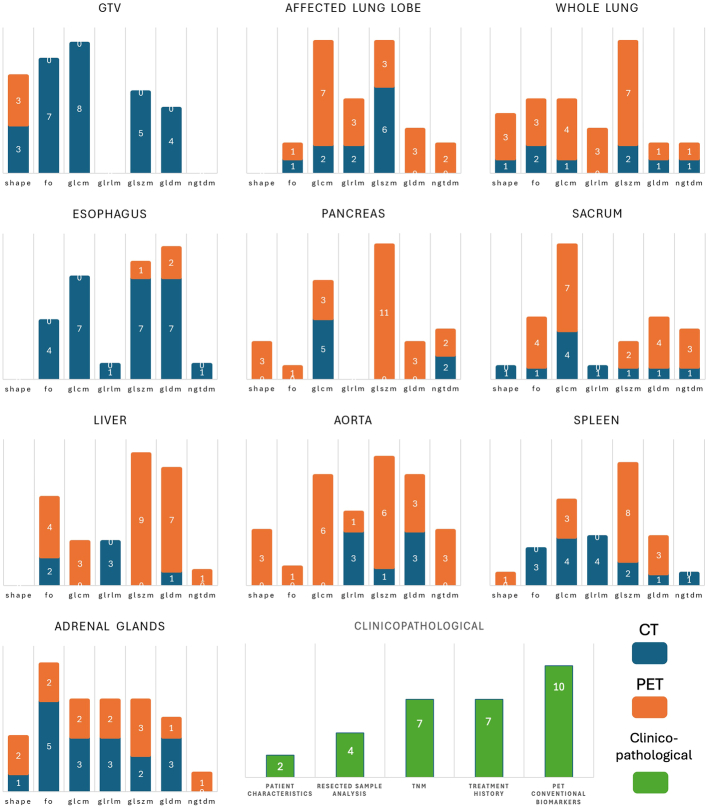
Number of features selected from each feature family in the first-level models. For the radiomics models, the number of features from CT and PET are shown separately.

Figure 7 shows the number of features selected from each feature family in the first-level models. Ten features were selected in each training fold (3-fold cross-validation) resulting in a total of 30 features for each first-level model. In the clinicopathologic model, conventional quantitative PET biomarkers had the highest frequency with 10 repetitions, and patients’ characteristics with 2 repetitions were least frequent. For the radiomic feature sets, the frequency of feature families is shown separately for CT and PET images. In GTV feature set, most features were from CT images and the GLCM family. In the affected lung lobe and the whole lung, PET features were also mainly present. In the organomics models, as a general trend features were selected from both imaging modalities. Among the radiomics features families shape features were the least and textures were the most selected ones.

**FIGURE 8:**
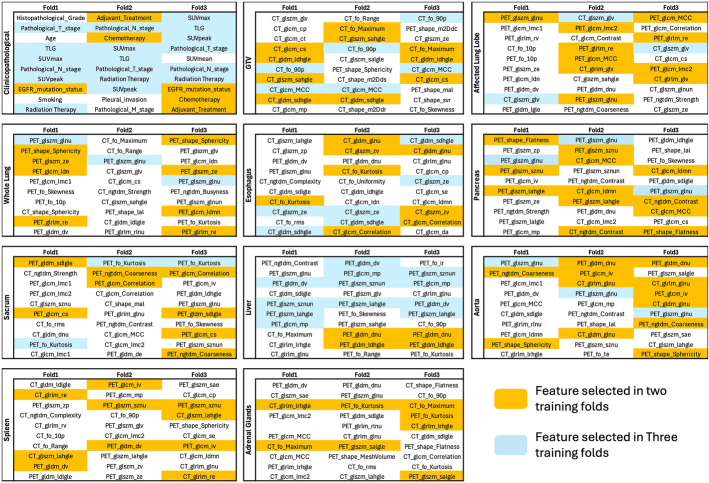
The full list of the selected features in the selected first-level models, separately for the 3 folds. Features that are selected in 2 and 3 training folds are color-coded.

Figure 8 shows the full list of the selected features for the first-level models, separately for the 3 folds. The features selected in 2 and 3 training folds are highlighted. In the clinical model, pathologic_T_stage, pathologic_N_stage, TLG, SUV_max_, SUV_peak,_ and radiation treatment were selected in all 3 folds. In the GTV model, 2 features from the CT images, including the first order 90 percentile (fo_90p) and maximal correlation coefficient (MCC) from the GLCM (glcm_mcc), were selected in all the folds. In the organomic models, as a general trend, PET features were more frequently selected in the folds than CT features.

## DISCUSSION

Non–small cell lung cancer (NSCLC) is now recognized as a heterogeneous disease with diverse clinical, histopathologic, and genetic traits, necessitating personalized treatment approaches.^10^ To achieve a holistic understanding of the patient’s disease, a wide range of biomarkers, including genetic status, clinical characteristics, histopathologic information, and imaging biomarkers must be integrated. In this study, we presented metapredictomics framework, which combines information from multiple domains using a modular ensemble learning strategy. We utilized metapredictomics framework to integrate clinicopathologic data with tumor radiomics and organomics^33^ information aiming to provide a more comprehensive view of the NSCLC disease and enhance the accuracy of recurrence prediction. A particular focus of the study was the incorporation of organomics, a novel radiomic approach that extracts features from multiple nontumoral organs. We hypothesize that the anatomic information of organs captured by CT and the metabolic activity mapped by FDG-PET may contain valuable insights into the progression of the disease within these organs or reflect the overall health of organs, both of which could influence the patient’s prognosis.

Several studies have investigated radiomics for NSCLC recurrence prediction. Nevertheless, they are mostly limited to features extracted from the tumor or its peripherals.^3,22,27,28^ Only few radiomics studies have explored beyond the primary lesion.^38^ Christie et al^39^ integrated patients clinical data with FDG-PET and CT radiomic features extracted from both the tumor and peritumoral regions, as well as radiomic features from the L3 to L5 vertebral bone marrow on FDG-PET, to improve recurrence risk stratification in patients with resectable NSCLC. Their proposed model achieved a concordance index of 0.76 and significantly outperformed a baseline model based solely on staging information. To identify advanced-stage lung cancer patients who will benefit from immunotherapy, Liao et al^40^ developed a progression-free-survival prediction model by integrating clinical and imaging biomarkers. They utilized intratumoral and peritumoral-vasculature radiomics from pretreatment CT images, and demonstrated enhanced prediction performance.^40^ To predict postradiation therapy complications, Guo et al^41^ utilized CT-based radiomics and dosiomics of not only the tumor GTV, but also the whole esophagus, reporting improved prediction performance when including the whole-esophagus features.

The performance of the first-level models was analyzed from 2 perspectives; first, we evaluated their standalone performance, reported in Table 3; second, we assessed their performance in combination with other models within our stacked ensemble models. The second evaluation was conducted by examining the frequency of each model’s appearance in the top 100 meta models (Fig. 5). Among the first-level models, the clinicopathologic model achieved the best results, with a C-index of 0.67 and appearing in 98 of the top 100 meta models. This highlights the importance of conventional biomarkers and suggests that radiomics technology would serve the best as complementary source of information beside conventional biomarkers, rather than fully replacing them. Following the clinicopathologic model, the GTV model with a C-index of 0.65 achieved close performance, as expected. Comparing the performance of affected lung lobe and whole lung models, the whole lung demonstrated superior performance (C-index: 0.65 vs 0.63) and was present more frequently in the top 100 meta models (71 vs 47, Fig. 5). This may be attributed to some lesions extending beyond a single lung lobe, while in the affected lung lobe model, we only selected the lobe containing the largest portion of the GTV as the ROI.

Mediastinal lymph nodes, lungs, brain, bones, liver, and adrenal glands have been introduced as preferential sites of recurrence in NSCLC.^14^ In our study, 12 organomics model achieved a C-index >0.6. Whole lung (C-index: 0.65), adrenal glands (C-index: 0.63), liver (C-index: 0.62), and sacrum (C-index: 0.62) models demonstrated strong prognostic performance both as standalone models and in combination with other models in the meta models (Fig. 5). The performance of the whole lung and aorta model matched the GTV model with a C-index of 0.65, followed by the esophagus and adrenal gland models with C-index values of 0.63. The aorta and esophagus can be sites of metastasis and recurrence due to their anatomic proximity to the mediastinum, where advanced NSCLC often spreads. The mechanism of the disease spread to these organs might be direct tumor extension, lymphatic spread, or hematogenous.^42,43^ The adrenal glands are a common site of metastasis in NSCLC due to their rich blood supply and proximity to the lungs. Their high vascularization and lymphatic drainage from the thoracic region make them vulnerable to spread of cancer cells.^44^ Our findings highlight the importance of maximizing the utilization of medical images by integrating information from various organs, rather than solely focusing the lens on the lesion or its surrounding environment. Furthermore, the impact of incorporating information from multiple sources was particularly evident in the meta-models, where many organomics models appeared in the top 100 meta-models. The best meta model demonstrated a significant performance increase, achieving a C-index of 0.731. It is notable that this significantly improved performance was not limited to the few best handpicked models and the top 100 meta models ranging from C-index of 0.703 to 0.731, all significantly outperforming the best standalone models (Fig. 4).

In Figures 7 and 8, we analyzed the selected features in the first-level models. Features were often selected from all feature families and from both PET and CT modalities, further emphasizing the importance of integrating multiple sources of information in model construction. In Figure 7, we present the selected features for each model, while color coding features were selected in more than one training fold. We believe that features selected across multiple training folds are potentially more reliable and should be further analyzed for their robustness and reproducibility in future studies. Among the clinical features, pathologic T and N stage and PET metrics, such as SUV_max_, SUV_peak_, and TLG were selected in all 3 folds. All features have previously been reported as predictive biomarkers for NSCLC recurrence.^13–15,45^ The treatment history of the patient also showed importance with radiation therapy being selected in all 3 and chemotherapy and adjuvant treatment in 2 folds, which highlights the importance of accurate selection of treatment regimens for the patients. The EGFR mutation status was also presented in 2 folds while the KRAS mutation status was not selected.

In the GTV radiomics model, 2 CT features including the fo_90p and the glcm_mcc were selected in all 3 folds. The fo_90p from the CT tumor region csaptures the upper range of tissue densities, often reflecting dense tumor components, such as viable tumor with high cellularity, fibrosis, or stromal tissue. These characteristics may reflect aggressive tumor biology and have been associated with increased recurrence risk in NSCLC.^46^ The glcm_mcc is a measure of the textural complexity in the ROI, which might be reflecting tumors with higher heterogeneity. This is in line with previous literature that demonstrated tumor heterogeneity is associated with progression and treatment failure.^39,47^ The GLSZM gray-level nonuniformity (glszm_glnu) extracted from the whole-lung region on FDG-PET was consistently selected across all folds. This feature quantifies variability in FDG uptake across zones within the lungs and may reflect global metabolic heterogeneity. Such heterogeneity could indicate occult tumor spread, inflammation, or systemic tumor-related effects, all of which are associated with an increased risk of recurrence in NSCLC.^22^ In the liver model, several PET features, including GLDM dependence variance (gldm_dv), GLSZM small zone nonuniformity normalized (glszm_sznun), and large area high gray-level emphasis (glszm_lahgle), were found to be significantly associated with recurrence risk. These features capture distinct aspects of metabolic texture, such as variability in local uptake patterns (gldm_dv), heterogeneity of small uptake zones (glszm_sznun), and the presence of large, high-uptake regions (glszm_lahgle). As the liver is typically a metabolically stable organ,^48^ deviations from this uniform uptake may reflect systemic metabolic alterations, inflammatory or physiological stress responses, or metastatic involvement, all of which are factors associated with poorer prognosis in NSCLC.^49,50^

An essential component of our framework is organ segmentation. Without an automated segmentation tool, the proposed approach would be impractical in clinical setting, as manual delineation of multiple organs per patient would impose a significant time and workload burden on clinicians. To address this issue, we employed our previously developed open-source CT-based organ auto-segmentation tool.^35^ This also helps reducing interobserver and intraobserver variability arising from differences between the users. For tumor segmentation, manually annotated masks were used. We acknowledge this as a limitation of the study, as all tumor segmentations were performed by a single radiologist, and no formal interobserver variability assessment was conducted.

To enhance the reproducibility, we applied standardized preprocessing steps in both the image and feature domains to enhance features reproducibility. However, specific feature harmonization was not considered.^51^ Given the relatively small data set and the limited number of patients per scanner type, applying harmonization techniques, such as ComBat, was not feasible. Future studies with larger and more balanced multiscanner cohorts may benefit from incorporating harmonization strategies. For the time-to-event analysis, we selected a simple and well-established model, ensuring that all clinicopathologic and radiomics models were constructed using the same framework to allow for fair comparison. In the second step, we applied a stacked ensemble technique by using the risk predictions from the first-level models as input for the meta model. This modular approach enables the creation of different stacked models optimized for various subsets of first-level models, allowing flexibility to accommodate patients with different information sources.

Unfortunately, due to the retrospective nature of the study, which did not include information regarding the recurrence site/organ, our analysis was limited in scope. We were unable to evaluate the prognostic power of our models from a recurrence site perspective, that is, we could not determine whether patients labeled as high-risk by a specific organ model later developed recurrence in that same organ. Another limitation was that although the brain is a common site of NSCLC recurrence, it was not included in our study because, in some patients, the PET/CT scans did not fully cover the brain. We recommend further studies with more detailed follow-up plans, including recurrence site data, to better investigate the potential of organomics approach and its interrelationship with other biomarker sources. A major limitation of our study was the size of the data set and its depth of information regarding the recurrence event. Future studies utilizing larger and more diverse data sets could further explore the frameworks proposed here, leading to more valuable and reproducible findings. A limitation of this study is its focus on a specific NSCLC cohort with PET/CT imaging, which may affect the generalizability of the findings to other NSCLC subtypes or imaging modalities. Furthermore, although this work focused on imaging and clinicopathologic predictors, integrating genomic or proteomic data could further refine prognostic modeling and should be explored in future studies.

## CONCLUSIONS

Our findings highlighted the importance of maximizing the utilization of medical images by utilizing information from various organs, rather than solely focusing the lens on the lesion or its surrounding environment. Furthermore, the impact of incorporating information from multiple sources was particularly evident in the meta models, where the integration of the outcome of clinicopathologic signature with primary tumor radiomics and various organomics models showed significant improvement in recurrence prediction.

## Supplementary Material
