# Supplementary material for: MetaPredictomics: A Comprehensive Approach to Predict Postsurgical Non–Small Cell Lung Cancer Recurrence Using Clinicopathologic, Radiomics, and Organomics Data
Source: Clin Nucl Med. 2025 Sep 3;50(12):1130–43. doi: 10.1097/RLU.0000000000006086 (PMC12617314; doi:10.1097/RLU.0000000000006086)
Supplement: Supplementary file 1 [file rlu-50-1130-s001.pdf]

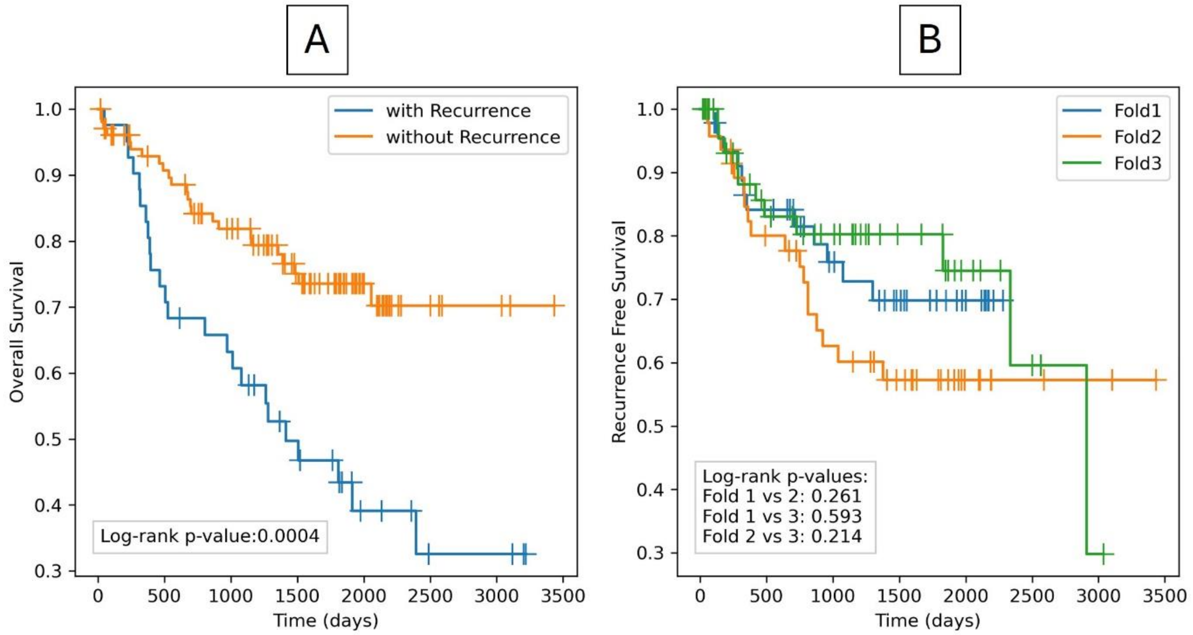

Supplementary Figure 1. A) The Kaplan Meier curves of overall survival of the patients with and without recurrence and the log-rank test p-value between the groups. B) Recurrence-free-survival (RFS) Kaplan Meier curves of the three dataset folds and log-rank tests p-values between the groups.

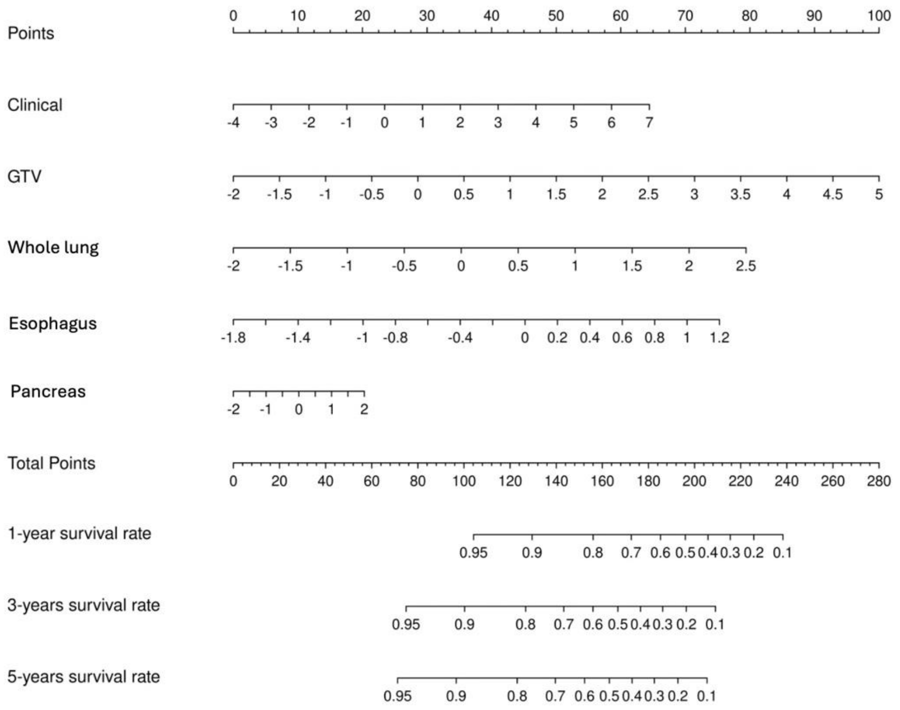

Supplementary Figure 2. Nomogram generated from the Meta#1 model (model with highest performance) for individualized prediction of 1-, 3-, and 5-year recurrence.

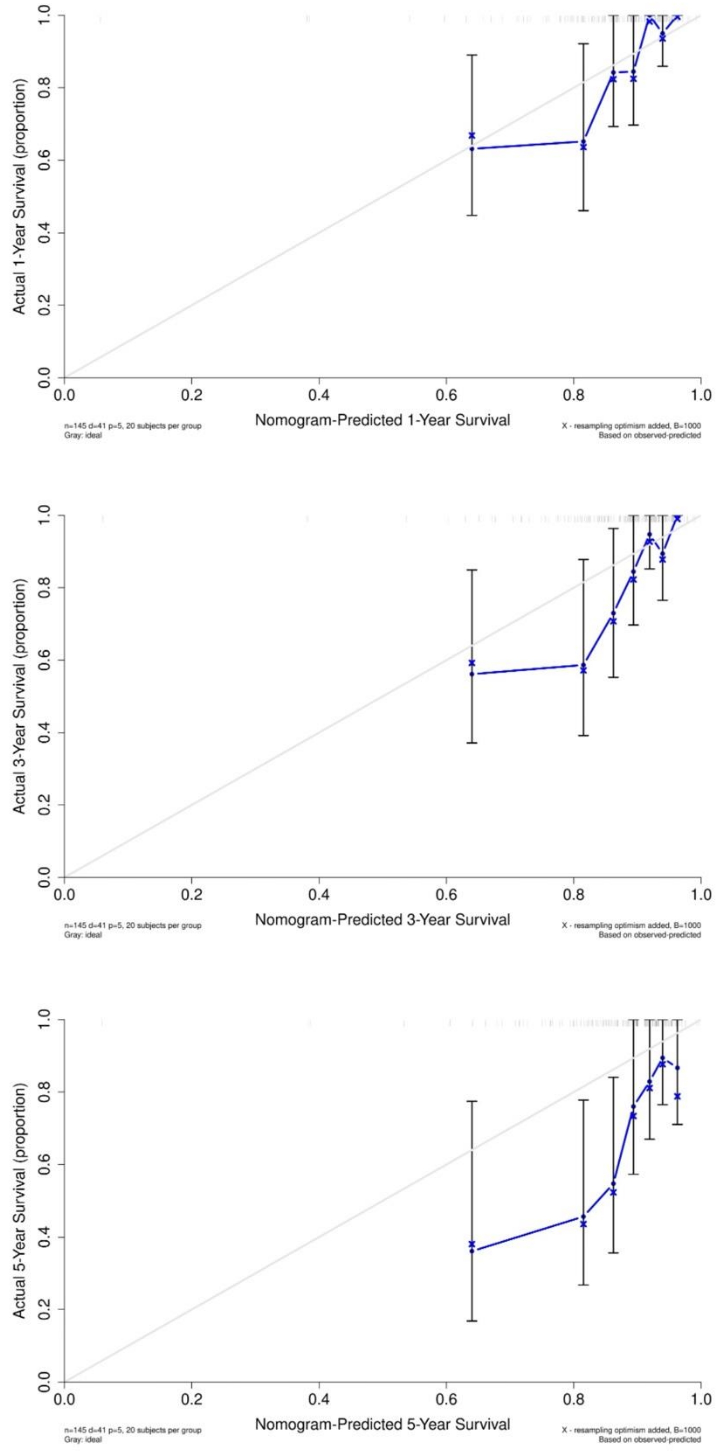

Supplementary Figure 3. Calibration plots for 1-, 3-, and 5-year recurrence predictions (from top to bottom). The calibration curves assess agreement between predicted and observed recurrence probabilities for the Meta#1 model. Patients were grouped into five quantiles based on predicted risk. The x-axis shows predicted recurrence from the nomogram, and the y-axis shows actual recurrence probability observed. The gray diagonal line represents perfect calibration. Blue points and error bars reflect the mean predicted and observed values with 95% confidence intervals, based on 1,000 bootstrap resamplings.
